# Quercetin Derivatives in Combating Spinal Cord Injury: A Mechanistic and Systematic Review

**DOI:** 10.3390/life12121960

**Published:** 2022-11-23

**Authors:** Sajad Fakhri, Mohammad Mehdi Gravandi, Sadaf Abdian, Seyed Zachariah Moradi, Javier Echeverría

**Affiliations:** 1 Pharmaceutical Sciences Research Center, Health Institute, Kermanshah University of Medical Sciences, Kermanshah 6734667149, Iran; 2 Student Research Committee, Kermanshah University of Medical Sciences, Kermanshah 6734667149, Iran; 3 Medical Biology Research Center, Health Technology Institute, Kermanshah University of Medical Sciences, Kermanshah 6734667149, Iran; 4 Departamento de Ciencias del Ambiente, Facultad de Química y Biología, Universidad de Santiago de Chile, Santiago 9170022, Chile

**Keywords:** quercetin, spinal cord injury, inflammation, apoptosis, oxidative stress, pharmacology

## Abstract

Spinal cord injury (SCI) possesses a complicated etiology. There is no FDA-approved treatment for SCI, and the majority of current interventions focus on reducing symptoms. During SCI, inflammation, oxidative stress, apoptosis, and autophagy are behind the secondary phase of SCI and cause serious consequences. It urges the need for providing multi-targeting agents, that possess lower side effects and higher efficacy. The plant secondary metabolites are multi-targeting agents and seem to provide new roads in combating diseases. Flavonoids are phytochemicals of continual interest to scientists in combating neurodegenerative diseases (NDDs). Flavonoids are being studied for their biological and pharmacological effects, particularly as antioxidants, anti-inflammatory agents, anti-apoptotic, and autophagy regulators. Quercetin is one of the most well-known flavonols known for its preventative and therapeutic properties. It is a naturally occurring bioactive flavonoid that has recently received a lot of attention for its beneficial effects on NDDs. Several preclinical evidence demonstrated its neuroprotective effects. In this systematic review, we aimed at providing the biological activities of quercetin and related derivatives against SCI. Detailed neuroprotective mechanisms of quercetin derivatives are also highlighted in combating SCI.

## 1. Introduction

Spinal cord injury (SCI) is a devastating irreversible neurological process consequence of high morbidity that can affect various aspects of patient life. As a serious injury to the nervous system, SCI result from a fall, a shooting, or an accident [1]. SCI is diagnosed at an annual rate of 10.4–83 per million people worldwide [2]. Two stages are behind SCI-associated pathological processes; the primary and secondary injuries. Following a sudden trauma, spinal fracture, or vertebral dislocation, primary injury begins [3]. Following the primary SCI, magnetic resonance imaging scans have revealed spinal cord swelling, cytotoxic edema, and hemorrhage [4]. Following the primary injury, oxidative stress, inflammation, apoptosis, autophagy, and mitochondrial dysfunction [5], are triggered by the secondary injury. Because the primary injury is a one-time and irreversible pathway, the secondary SCI has become a golden time for treatment to stop further damage. Since no effective treatment has been approved for treating SCI, providing novel compounds to suppress inflammation, apoptosis, and oxidative stress while increasing autophagy is of great importance. Neuronal atrophy, synaptic abnormalities, and axon regeneration are also the main dysregulated events impeded by glial scar formation in SCI [6].

Phytochemicals are a critical candidate in modulating chronic diseases such as cancer, diabetes, and cardiovascular disease [7,8]. Polyphenols can be found in fruits, vegetables, and beverages derived from plants, are micro-constituents. Over the past three decades, a lot of research has been conducted on plant-derived phenolic compounds. Growing reports are showing the protective effects of polyphenols on major diseases such as cardiovascular disease [9], cancer [10], diabetes [11], obesity, and viral infections [12,13]. The potential role of polyphenols in modulating neurological disorders has been the focus of ongoing intensive efforts [14].

In this line, polyphenols have opened promising avenues for developing new and effective pharmaceuticals. The most diverse class of phytochemicals are flavonoids, which are abundant in higher plants and have the tremendous therapeutic potential [8]. Based on their chemical skeleton, flavonoids are categorized into six classes, including isoflavonoids and anthocyanidins, as well as flavanols, flavanones, flavones, and flavonols [15]. Flavonoids have shown a prospect in preventing/treatment of neurodegenerative diseases (NDDs) through multiple mechanisms [16].

Quercetin is one of the predominant flavonoids that are more frequently found in edible plants and is one of the most potent antioxidants of plant origin [17]. Flavonols are a major class of polyphenols known as flavonoids [18,19]. As an anti-inflammatory, anti-carcinogenic, anti-infective, and antioxidant flavonol, quercetin has shown numerous biological activities and beneficial effects on human health. It also stimulates mitochondrial biogenesis and prevents platelet aggregation and lipid peroxidation [20]. Previously, the neuroprotective effects of quercetin have been investigated in NDDs such as cognitive impairment [21], ischemia, traumatic injury [22], Parkinson’s disease (PD) [23], and Huntington’s disease (HD) [24]. We have previously provided the critical role of polyphenols against SCI complications. Quercetin showed neuroprotective activities and significantly promoted functional recovery following SCI via targeting p38 mitogen-activated protein kinase (MAPK)/inducible nitric oxide synthase (iNOS), NLR family pyrin domain containing 3 (NLRP3), NF-E2-related factor 2 (Nrf2)/Keap1/antioxidant response element (ARE), and phosphoinositide 3-kinases (PI3K)/protein kinase B (Akt)/mammalian target of rapamycin (mTOR) signaling pathways [25,26,27,28,29,30,31]. A recent report has also highlighted the mTOR-mediated neuroprotective roles of quercetin in combating SCI [32]. To the best of our knowledge, this is the first systematic review providing biological activities and pharmacological mechanisms of quercetin derivatives against SCI. Additionally, the whole pharmacological mechanisms of quercetin complexes (e.g., quercetin, rutin, quercetin-3-*O*-glucoside, quercetin-3-*O*-glucoronide, and luteolin) have been revealed against SCI.

## 2. Materials and Methods

**Figure 1:**
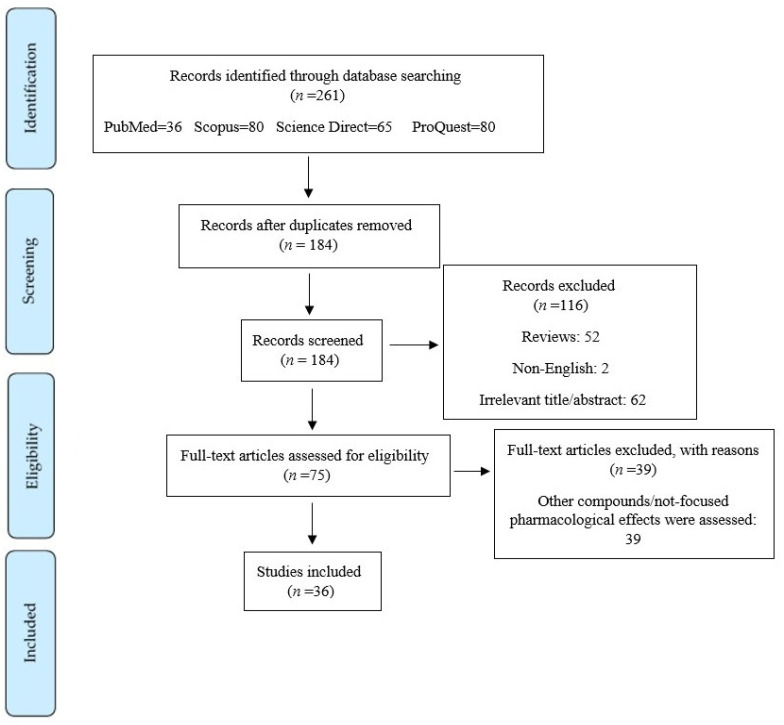
PRISMA flowchart on the literature search process.

Based on the PubMed, Web of Science, Proquest, and Scopus databases, a comprehensive and systematic review was conducted. The keywords (“spinal cord injury”) AND (“quercetin” OR “3-hydroxyflavone” OR “isoquercetin” OR “hyperoside” OR “rutin” OR “rhamnetin” OR “isorhamnetin” OR “solophenol” OR “uralenol” OR “quercetin 3, 7, 3’, 4’ tetrasulfate” OR “icaritin” OR “rhamnazin” OR “isorhamnetin-3-*O*-glucoside” OR “doramannin” OR “dihydroquercetin” OR “quercetin glucuronide” OR “Quercetin pentaacetate” OR “Quercetin 7-*O*-paramethyl” OR “Quercetin7-*O*-paranitro” OR “7-*O*-paramethylbenzyl quercetin complex” OR “7-*O*-paranitrobenzyl quercetin complex”) were searched in [All fields]. From the beginning to October 2022, data were collected, with an emphasis on English-language papers. M.M.G. and S.Z.M., two independent researchers, carried out the search screening. As a result, any disagreements were discussed with the first author (S.F.) and resolved. A systematic search of electronic databases yielded 261 articles, but 77 were left out due to duplication and reviews, respectively. In addition, title/abstract, and full text of 62, and 39 articles indicated that they were irrelevant. The systematic portion of this review included 36 reports after excluding non-relevant full texts (Figure 1).

## 3. Results and Discussion

### 3.1. Biological Sources, Chemical Structure, and Pharmacokinetics of Quercetin

**Figure 2:**
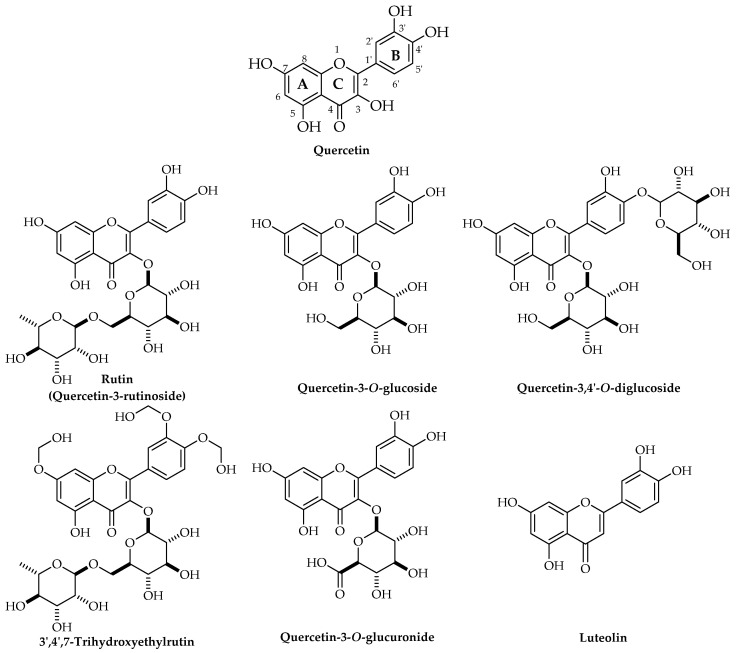
Chemical structures of quercetin and its major derivatives.

In comparison with other phytochemicals, quercetin is an abundant flavonol [33]. Quercetin includes five hydroxyl (OH) groups which are located at 3, 3′, 5, 7, and 4′ positions (Figure 2). The replacement of the OH group with a glycosyl group leads to the production of quercetin glycoside from aglycone form [34]. 

Quercetin solubility is high in lipids and alcohol, while this compound is quite water-insoluble [35]. Due to the presence of a glycosyl group, quercetin glycoside is water soluble. There is a complicated metabolic pathway behind quercetin. Both glycosidic and aglycone forms could be absorbed by passive diffusion or via organic ion transporting polypeptide through the small intestine [36]. The liver, intestine, and kidneys are responsible for quercetin metabolism. There is a low bioavailability (0–50%) and short half-life (1–2 h) for quercetin in humans [37]. According to the structure-activity relationship (SAR), quercetin possesses a free radical neutralizing potential for combating NDDs [38]. Considering quercetin SAR, has also shown features of antioxidation, and anti-inflammation, inhibiting apoptosis and promoting autophagy, towards improving pathological changes in SCI [12,13,32]. Multiple studies have demonstrated that the microenvironment and cell survival after SCI can be improved by regulating mTOR, nuclear factor-kappa B (NF-κB), MAPK, and PI3K [39].

In vitro cell culture experiments demonstrated that OH substitution positioned at C5 and C7; C3 positioned at methoxy (OCH_3_) or H substitution, and OH/OCH_3_ substitution at the C4 position improved the neuroprotective properties of phenolic compounds. The B- ring’s *ortho*-dihydroxy substitution, C-ring’s 4-carbonyl, and A-ring’s 5 or 3 hydroxy substitutions proved quercetin ion chelating activity, and reduced hydrogen peroxide (H_2_O_2_)-induced calcium (Ca^2+^) dysregulation and oxidative stress [40]. The OH groups in the C-ring and A-ring are important for the neuroprotective activities of quercetin. 

Antioxidant, anti-inflammatory, and anti-apoptotic properties are important biological features of quercetin that make this compound attractive for controlling NDDs [41].

### 3.2. Neuroprotective Potentials of Quercetin Derivatives

Quercetin’s neuroprotective effects have been extensively researched. It prevents neuronal oxidative stress-induced cell toxicity at low micromolar concentrations. Quercetin also stimulates neuronal regeneration while suppressing neuroinflammation by inhibiting inflammatory mediators such as iNOS and NF-кB. Quercetin active metabolites (e.g., glucuronidated, methylated, or sulfated) have also shown neuroprotective responses, in preclinical models [42,43,44]. Quercetin showed protective roles against mitochondria and dopaminergic neurons through the PKD1-Akt signaling pathway in both in vivo and in vitro models of PD [45].

Quercetin is effective as a treatment for Alzheimer’s disease (AD), enhancing learning, memory, and cognitive functions [46]. The OH groups in quercetin strongly interact with the acetylcholinesterase (AChE) enzyme at the catalytic anionic site through hydrogen bonding. Quercetin glycosylation at position 3 makes a decrease in AChE blockage potential as well as free radical scavenging activity [47]. This conjugation system is an essential factor for free radical scavenging activity and the beneficial effects of quercetin for managing oxidative stress in NDDs [38]. Wand and co-authors, in a mouse model of AD, evaluated the effects of long-term administration of quercetin on cognition and mitochondrial dysfunction. They noted that quercetin improved mitochondrial dysfunction by restoring ATP synthesis, lowering reactive oxygen species (ROS) production, and restoring mitochondrial membrane potential. Additionally, quercetin increased the expression of AMP-activated protein kinase (AMPK), a vital energy metabolism regulator in cells [48]. Quercetin and rutin have also been shown to improve memory in zebrafish subjected to scopolamine-induced memory impairment. This suggests that quercetin derivatives may improve cholinergic neurotransmission [49]. Khan and coworkers [50] and Shimmyo et al. [51] concluded that the administration of quercetin inhibited AChE and secretase enzymes in vitro models, thereby preventing acetylcholine degradation and decreasing Aβ production [52]. Administration of quercetin nanoparticles significantly reduced neuronal degenerative changes, decreased the formation of amyloid plaques (APs) and neurofibrillary tangles (NFTs), and increased cellular proliferation. At molecular, cellular, and subcellular levels, quercetin nanoparticles reduced aluminum chloride-induced damaging effects on hippocampal neurons [53].

Electrical discharges, neurotransmitters, and associated ion channels are the main pathologies of epilepsy, so quercetin is targeted as the primary treatment. Through its effects on various cell types, quercetin has been shown to reduce seizures [54].

Sharma et al. concluded that the combination of quercetin and piperine improved the antioxidant, anti-inflammatory, and neuroprotective effects of PD. In experimental rats, quercetin reduced rotenone and iron supplement-induced motor deficits and also modulated biochemical and neurotransmitter changes. Nonetheless, a combination of quercetin with piperine upgraded its neuroprotective impact as contrasted and treatment with quercetin alone [55]. In addition, quercetin and its nanocrystals reduced the level of malondialdehyde (MDA) in the hippocampal area, prevented memory disruption in a 6-hydroxydopamine model of PD, and increased activities of antioxidant enzyme (e.g., catalase, glutathione, and superoxide dismutase) [56].

### 3.3. Pathophysiological Mechanisms of Spinal Cord Injury

SCI is accompanied by primary and secondary damage phases. The primary stage of SCI is initiated immediately after the injury following spinal ligament tearing and features of bone fragments [57,58,59]. Axonal disruption, glial membrane disruption, hemorrhage, and destruction of neural parenchyma comprise first-phase events. The primary injury facilitates secondary injury that leads to more mechanical and chemical damage to spinal tissues [1,57,58,59]. Also, enhancing concentrations and production of reactive oxygen, and increasing the accumulation and levels of glutamate and Ca^2+^ within cells promote neuronal excitotoxicity as well as phospholipids, proteins, and nucleic acid damage that cause neurological dysfunction. Typically, secondary phases of injury are classified into three phases: acute, chronic, and sub-acute [1,57,58,59]. Acute secondary injury is initiated following the primary phase that is accompanied by several clinical features, including vascular damage, necrosis, ionic imbalance, edema, excitotoxicity, inflammation, production of free radicals, lipid peroxidation, and increasing Ca^2+^ influx. Persists of acute secondary injury led to the beginning of sub-acute secondary injury that was specified by neuronal apoptosis, formation of the glial scar, axonal demyelination, axonal remodeling, and Wallerian degeneration [1,57,58,59]. Chronic secondary injury is the last phase of SCI that is described by the maturation of glial scar, axonal dieback, and cystic cavity formation. The pathophysiology of SCI comprises interrelated consecutive mechanisms and events. Simultaneously occurrence of multiple events leads to complicated attributes that make difficulties in the treatment process. Inflammatory responses, ROS, free radicals, N-methyl-D-aspartate (NMDA), excitatory amino acids, opiate receptors, and apoptosis-related signaling are some of the important mechanisms and parameters involved in the SCI process [57,58].

#### 3.3.1. Inflammatory Responses

Following SCI, an inflammatory response can be initiated with several peripheral immune cells such as T cells, neutrophils, and macrophages. Depending on the time and duration of inflammatory responses following SCI, these complex responses can produce neuroprotective and neurotoxic effects [57,58]. Meaningful beneficial functions from macrophages and other early inflammatory mediators and cells. The levels of variant inflammatory mediators such as platelet-activating factors, leukotrienes, serotonin, bradykinin, and prostaglandins enhanced in the lesion sites [57,58]. Pathological changes in microglia are subsequent inflammatory cytokines including interleukin (IL)-10, tumor necrosis factor (TNF), IL-6, and IL-1 that promote inflammatory reactions in the lesion sites [60,61]. Similarly, neutrophils and macrophages can facilitate tissue damage and lead to the growth of lesions. Furthermore, several cytokines, nitric oxide, chemokines, oxygen, as well as various nitrogen-containing molecules may induce the commencement of central nervous system inflammatory responses [57,58].

#### 3.3.2. ROS and Free Radicals

The generation of high levels of ROS and reactive nitrogen species (RNS) can lead to several damaging effects, such as lipid peroxidation in variant organs [58]. Production of ROS and free radicals exert a considerable role in the progression of variant disorders, including variant types of cancer, cardiovascular, AD, PD, and other NDDs [25,26,27,28,29,30,31,62,63,64,65]. Suppression of free radical’s generation is essential for enhancing the viability of cells. Reducing the activity of the endogenous antioxidant system exerts a pivotal impact on SCI. In several in vivo and in vitro studies, variant plant secondary metabolites, including curcumin, resveratrol, quercetin, and ginsenoside, as well as vitamin E, and selenium showed significant neuroprotective activities, and effective potentials in the management of SCI via diminishing lipid peroxidation, and alternating ROS production and free radicals [57,58].

#### 3.3.3. NMDA, Excitatory Amino Acids, and Opiate Receptors

One of the important and known excitatory neurotransmitters in the central nervous system is glutamate and the overactivation of glutamate receptors leads to neuronal damage. The levels and activity of excitatory amino acids such as aspartate and glutamate enhanced shortly following SCI. NMDA, AMPA, metabotropic glutamate, and kainate receptors are four major known classes of glutamate biochemical receptors that regulate the entry of Na^+^, K^+^, and Ca^2+^. NMDA receptors are a family of L-glutamate receptors that play a considerable role in memory, learning, and spatial memory [57,58,66]. Excitatory neurotransmitter exerts direct influence in the spinal cord by NMDA receptors and blocking these receptors lead to significant protection against secondary damage owing to ischemia and trauma. Antagonists of NMDA can remarkably reduce the incidence of edema and improve neurological functions [57,58,67]. Also, ion channels of the NMDA receptors can be blocked by magnesium ions. The lipid peroxidation process can be suppressed by magnesium ions through antagonizing glutamate receptors. It was reported that the administration of α-amino-3-hydroxy-5-methyl-4-isoxazolepropionic acid antagonists leads to improvement of function and decreases the injured area [57,58]. Moreover, it was reported that the local release of opioid peptides was elevated during SCI which makes more strengthens this hypothesis that endogenous opioids may have substantial effects on neuronal damage and secondary injury as blocking opiate receptors lead to protecting the neuronal cell against damage via suppression release of cellular contents [68]. Also, it was demonstrated that Ca^2+^ channel blockers agents can notably prevent secondary SCI due to the role of Ca^2+^ ions in cell death, and activating the phosphorylase, proteases, and phospholipase in cells [69]. Similarly, administering naloxone, a non-selective antagonist, lead to improving clinical outcomes and blood flow following acute SCI [57,58,66].

#### 3.3.4. Apoptosis

Free radicals and the release of inflammatory cytokines following SCI facilitate apoptosis leading to excitotoxicity and inflammation. After 3 h to 8 weeks following SCI in the areas surrounding the injured spinal cord tissue apoptosis occurs. Moreover, neuronal demyelination resulting from apoptosis of oligodendrocytes appears after a few weeks of injury [70,71,72]. Oligodendrocyte changes are one of the characteristics of SCI. Variant previous studies emphasized that apoptosis promotes secondary inflammatory damage via increasing neuronal loss and deterioration of the microglia [57,58]. Caspases are cysteine proteases that promote inflammation and programmed cell death. Following SCI, the levels and activity of several caspases are enhanced via activation of their components that lead to inducing apoptosis in particular cells, such as neurons, microglia, oligodendrocytes, and astrocytes. Due to the inability of spinal cord neuron cells to reproduce, the concern about recovery and treatment of patients with SCI is crucial [57,58]. Contrariwise, glial cells have a meaningful ability to divide and regenerate that can elevate neuronal protection via two mechanisms [70,71,72]. The central potentials of glial cells to metabolic and neurotrophic support of neurons promote recovery of the injured neurons. Similarly, scavenging apoptotic mediators such as cytokines, and suppression of free radicals leak from dying neuronal cells are reported as the second neuroprotective mechanism of glial cells to protect injured neurons in SCI [70,71,72].

#### 3.3.5. Local Vascular Effects

Reducing blood supply is reported as one of the substantial consequences of severe SCI that lead to the initiation of ischemia. Disruption in autoregulation of the spinal cord facilitates abnormal alteration in systemic hemodynamics, systemic hypotension, and hypoxia that can reduce blood flow in the spinal cord and exacerbate ischemia [57,58]. Moreover, due to ischemia, the generation of ATP, transportation, and supply the oxygen and glucose to tissues diminish [73]. Focal narrowing of sulcal arterioles, aneurysmal dilatation, occlusion, or fragmentation is suggested as one of the reasons for post-traumatic ischemia. Endothelium capillary damage, accumulation of vasoactive cytokines, and congestive compounds, including fibrin and platelets, enhancing the lactic acidosis that leads to reducing pH of tissue are other factors that may have effects on the progression of ischemia after trauma [57,58]. The permeation of protein from damaged spinal cord veins causes edema around peripheral tissues and injured areas that led to enhancing the pressure of the spinal cord and disruption of blood flow. In addition, the concentration of electrolytes abnormally changes after spinal cord trauma, due to ischemia-reperfusion injury the levels and production of free radicals and glutamate elevate which can accelerate and intensify the devastation of the blood-spinal cord barrier [57,58].

**Figure 3:**
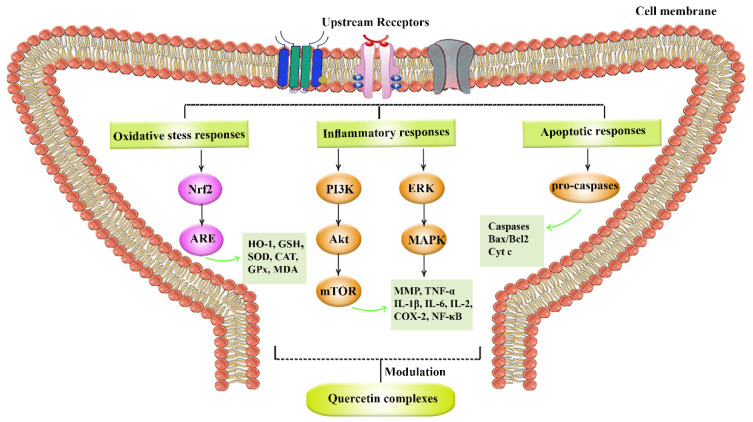
Major dysregulated pathways behind SCI. Akt: protein kinase B, ARE: antioxidant response elements, CAT: catalase, COX: cyclooxygenase, Cyt c: cytochrome c, ERK: extracellular regulated kinase, GPx: glutathione peroxidase, GSH: glutathione, HO-1: heme oxygenase-1, IL: interleukin, MDA: malondialdehyde, MAPK: mitogen-activated protein kinase, MMP: matrix metalloproteinase, mTOR: mammalian target of rapamycin, NF-κB: nuclear factor-kappa B, Nrf2: NF-E2-related factor 2, PI3K: phosphoinositide 3-kinase, SOD: superoxide dismutase, TNF-α: tumor necrosis factor-alpha.

Figure 3 provides the complex pathophysiological mechanisms behind SCI.

### 3.4. Quercetin Derivatives against Spinal Cord Injury

Rutin as a flavonoid polyphenol and a quercetin derivative has shown antioxidant and anti-inflammatory activities [74]. Rutin prevents morphological changes by reducing the expression of p53 and apoptosis while increasing the enzymatic activity of endogenous antioxidants. Rutin significantly reduced reactive oxygen species NLRP3, MDA, IL-18, IL-1β, TNF-α, and caspase-1, caspase-3 and 9. It also reduced macrophage inflammatory protein-2 (MIP-2), matrix metalloproteinase-9 (MMP-9), and phosphorylation of p-Akt [74] in a rat model of SCI. It also blocked the p38MAPK pathway, thereby controlling inflammatory processes [75], as well as histological damages to improve motor recovery. Mild hypothermia plus rutin increased the rat’s BBB score; improved spinal cord tissue regeneration; and reduced TNF-α after SCI. This adjuvant therapy increased MPO; and reduced spinal cord MDA and ROS, indicating the anti-inflammatory and antioxidative stress mechanism of rutin in alleviating SCI complications [76,77]. The anti-nociceptive and neuroprotective responses of rutin-containing compounds have also been shown to pass through MAPK activation [78]. As another quercetin complex, trihydroxyethyl rutin showed neuroprotective responses, improving nerve electrophysiological parameters and limb motor function following SCI, maintaining microvascular density, and decreasing injury area and demyelination degree [79]. Overall, as a quercetin complex, rutin has shown promising anti-inflammatory and antioxidant effects in the modulation of SCI-induced sensory-motor dysfunction.

Quercetin is a flavonoid commonly found in food, and with a high concentration in onion, apple, tea, wine, and in varieties of Chinese herbs. It is an important flavonoid found, showing antioxidative properties and scavenging free radicals, anti-inflammatory, balancing apoptotic processes [80], interaction with important specific proteins of intracellular signaling cascades, and iron chelation [81,82]. The critical role and novel insights of quercetin have been shown in SCI [83,84]. Quercetin exerted antioxidant effects [85] by blocking p38MAPK/iNOS signaling pathway, downregulating MDA content, and upregulation of superoxide dismutase activity, which together inhibited secondary oxidation following SCI in rats [86,87]. Quercetin inhibited apoptosis by targeting the p38MAPK pathway in rats following SCI. In line, the anti-apoptotic activity of a common quercetin glycoside in onions, quercetin 3,4′-*O*-β-D-diglucoside, was applied through modulation of Bax/Bcl-2 ratio in human striatal precursor cells via nutrient deprivation. On the other side, hyperoside (3-*O*-galactoside of quercetin) exhibited protective effects against neuronal ischemia-reperfusion through suppression of extracellular-regulated kinase (ERK), c-Jun N-terminal Kinase (JNK) and Bcl-2 family-related apoptotic signaling pathways in rat cortical nerve cells. Quercetin also reduced the rate of nitric oxide and MDA while enhancing total antioxidant levels in rats with SCI injury [88,89].

Quercetin-3-*O*-glucuronide exhibits neuroprotective effects in human embryonic neural stem cells by enhancing Akt phosphorylation, cyclin D1 expression, and brain-derived neurotrophic factor production in a model of monosodium glutamate-induced excitotoxicity of spinal cord motoneurons [90]. Quercetin inhibited glial fibrillary acidic protein in the satellite glial cells of the bilateral L5 dorsal root ganglions (DRGs). In their study, quercetin suppressed the development of neuropathic pain through the inhibition of satellite glial cells in male Sprague-Dawley rats [91]. Quercetin also showed inhibitory effects on the production of IL-1, IL-6, TNF-α, and IL-8 in lipopolysaccharide-stimulated models of neuronal damage [92]. This flavonol compound meaningfully reduced necroptosis of oligodendrocytes after SCI and suppressed macrophages/microglia polarized to M1 phenotype through inhibition of signal transducer and activator of transcription 1 (STAT1) and NF-κB pathway in male Sprague-Dawley rats [93]. Quercetin also decreased the level of MPO which stimulated H_2_O_2_ causing cellular damage in adult male Wistar rats [94]. During a rat model of SCI, quercetin increased the 5-HT-positive nerve fibers, NF-200-positive neurons, and brain-derived neurotrophic factor (BDNF), while decreasing glial fibrillary acidic protein (GFAP) positive astrocytes, p-JNK2 and p-STAT3 production [95]. Quercetin blocked the production of GFAP, blocked the phosphorylation of Akt, mTOR, and p70S6K, and improved axonal regeneration after SCI [96]. In combination therapy of quercetin and human umbilical cord mesenchymal stromal cells (HUMSCs) beneficial impacts were shown on Sprague-Dawley (SD) female rats SCI models. Such effects were applied by decreasing inflammatory mediators such as IL-1β and IL-6 and the size of the cystic cavity, enhancing anti-inflammatory agents such as IL-4, IL-10, and transforming growth factor (TGF)-β1 [97]. Also, the combined administration of quercetin and bone marrow stromal cells (BMSC) showed better improvement in adult male Sprague-Dawley rats with SCI via increasing the expression of Cldn5, Ocln, and Tjp1, decreasing blood-spinal cord barrier, therefore, reducing inflammatory processes in neuronal cells, and lowering the production of NF-кB [98]. In line, the anti-neuroinflammatory effects of quercetin were shown in similar reports on SCI [99]. During another in-line report, in vivo neuroprotective mechanisms of rutin were shown to pass through anti-inflammatory, antioxidant and suppressing p38MAPK after SCI [100,101]. In similar models of neuropathic pain, quercetin also showed neuroprotective responses through anti-apoptotic pathways [102,103]. Histological and biochemical staining also confirmed the potential of resveratrol and quercetin in preventing secondary damage during SCI [102,104,105].

The combination of curcumin and quercetin showed beneficial effects in the treatment of SCI via decreasing serum S-100b levels and spinal cord tissue S-100b levels [106], reducing the activity of MAPK, and increasing Fe^2+^-chelation and Fe^2+^-clearance after SCI. The combination of curcumin and quercetin decreased a delay in Ca^2+^ deregulation, MDA, and phosphorylated-p38MAPK levels, reducing the reactivation of astrocyte and the activity of 6-hydroxydapamine, increasing the activity of catalase after traumatic SCI [107]. In an in vivo model of SCI, isoquercetin (quercetin-3-*O*-glucoside) improved synaptic plasticity and motor dysfunctions. Isoquercetin also modulated histopathological damages, reduced the fibrillization of α-synuclein, and hippocampal neuronal cell death, in vivo [108]. In all, quercetin has shown anti-inflammatory, anti-apoptotic, and antioxidant effects in combating SCI. To apply such neuroprotective effects, quercetin modulated multiple dysregulated mediators during the secondary phase of SCI.

As another derivative of quercetin, luteolin, a flavone compound gained from *Cissus quadrangularis* L. [Vitaceae], the blocked activity of caspase-1 and Rho-associated protein kinase 2 (ROCK2) that is involved in inflammatory processes of SCI [109]. Isorhamnetin, another quercetin derivative, promoted functional recovery in rats by activating Nrf2/heme oxygenase 1 (HO-1) pathway and thereby combating oxidative stress following SCI. Isorhamnetin also promoted M2 macrophage activation and suppressed the activation of microglial/glial and suppressed inflammatory cytokines including monocyte chemotactic protein-1 (MCP-1), TNF-α, and IL-1β [110].

Altogether, quercetin complexes have shown a bright future in the attenuation of dysregulated pathways after SCI. Accordingly, quercetin improved sensory and motor function, as well as, neuronal survival through multiple mechanisms, including anti-apoptosis, antioxidant and anti-inflammatory responses.

Table 1 provides the neuroprotective roles of quercetin derivatives in SCI.

## 4. Conclusions

Despite perspective progressions in treating NDDs, SCI remained a primary cause of disability and a global challenge. Oxidative stress, inflammation, apoptosis, and autophagy have shown crucial roles in the pathogenesis of SCI. In most studies, increased inflammation, oxidative stress, and apoptosis is associated with cell survival after SCI. Besides, preventing aforementioned dysregulated pathways has been demonstrated to play an impressive role in improving post-SCI complications. Such complicated mechanisms urge the need for finding novel multi-targeting agents. Phytochemicals are promising multi-targeting agents in combating NDDs and SCI.

Quercetin is a plant-derived flavonoid with antioxidative, anti-inflammatory, anti-apoptotic, and autophagy regulation [80] in NDDs [81]. Quercetin and its derivatives promoted the process of neuronal cell regeneration in SCI via the attenuation of several dysregulated pathways. Quercetin derivatives showed the potential of blocking the p38MAPK/iNOS signaling pathway [86], suppressing the production of inflammatory mediators such as IL-1, TNF-α, IL-6, and IL-8 [92], and increasing the 5-HT-positive nerve fibers [106], and brain-derived neurotrophic factor (BDNF). Quercetin derivatives decreased the production of NF-кB [98], myeloperoxidase [94], GFAP positive astrocytes, p-JNK2 and p-STAT3 production [95], serum S-100b levels [106], and reducing reactivation of astrocyte and the activity of 6-hydroxydopamine [107] during SCI. Additionally, ongoing reports are reporting novel strategies towards regeneration, including the application of novel delivery systems [112] and targeting senescent cells by natural products for SCI [113] (Figure 3).

Future reports should include extensive in vitro and in vivo experimentation to reveal precise signaling pathways followed by well-controlled clinical trials to assess the potential of quercetin derivatives against SCI. Such research will highlight more potential applications of quercetin derivatives in the prevention, management, and treatment of SCI.

## Figures and Tables

**Table 1:** Neuroprotective mechanisms of quercetin derivatives in spinal cord injury.

Quercetin and Derivatives	Study Type	Cell Line/Animal Type	Mechanism	Reference
Quercetin	In vivo	Sprague-Dawley rats	↓ROS, ↓NLRP3, ↓TNF-α, ↓IL-1β	[111]
			↓neuropathic pain, ↓satellite glial cells, ↑ability to walk, ↑Cldn5, ↑Ocln, ↑Tjp1	[81,87,98]
			↑SOD, ↑GSH, ↓polymorphonuclearleukocyte infiltration,	[85]
			↓iNOS, ↓p38MAPK, ↑SOD, ↑MDA	[86]
			↓MDA, ↓NO	[112]
			↓GFAP, ↓neuropathic pain	[91]
			↓TNF-α, ↓IL-1β, ↓IL-6, ↓ IL-8	[92]
			↓STAT1, ↓NF-κB	[93]
			↓MPO	[94]
			↑5-HT-positvie nerve fibers, ↑BDNF	[95]
			↓GFAP, ↓phosphorylation of Akt, ↓mTOR,	[96]
			↑IL-4, ↑IL-10, ↑TGF, ↓IL-6, ↓IL-1	[97]
		C57BL/6J mice	↑neuronal intrinsic growth capacity, ↑functional recovery,	[80]
		Wistar albino rats	↓MDA, ↓NO, ↓caspase-3, ↑SOD, ↑GSH	[89]
Quercetin 3,4′-*O*-β-D-diglucoside	In vivo	Sprague-Dawley rats	↓MDA, ↓NO, ↑total antioxidant levels	[88]
Quercetin-3-*O*-glucuronide	In vivo	Sprague-Dawley rats	↓MDA, ↓IL-1, ↓IL-6, ↓TNFα, ↓INFɣ, ↓caspase-3 activity, ↑SOD, ↓p38 MAPK	[90]
Rutin (Quercetin-3-Rutinoside)	In vivo	Sprague-Dawley rats	↑MAPK	[78]
			↓MIP-2, ↓MMP-9, ↓p Akt, ↓p38MAPK	[74]
			↓cell death, ↓IL-1β, ↓TNF-α, ↓p38MAPK	[75]
			↓TGF-β/Smad,	[76]
			↓MPO, ↓MDA, ↓ROS, ↓TNF-α	[77]
3′,4′,7-trihydroxyethylrutin	In vivo	Sprague-Dawley rats	↓size of coronal, sagittal, and transversal lesions	[79]
Combination of quercetin and curcumin	In vivo	Wistar albino rats	↓S-100β, ↓p38MAPK, ↑Fe^2+^-chelation, ↑Fe^2+^-clearance, ↓6-OHDA, ↑CAT	[106,107]
Luteolin	In vivo	Wistar albino rats	↓activity of caspase-1, ↓ROCK2	[109]

BDNF: brain-derived neurotrophic factor, CAT: catalase, GFAP: glial fibrillary acidic protein, GSH: glutathione, IL: interleukin, INFɣ: Interferon-gamma, iNOS: inducible nitric oxide synthase, MDA: malondialdehyde, MIP: macrophage inflammatory protein, MMP: matrix metallopeptidase, MAPK: mitogen-activated protein kinases, MPO: myeloperoxidase, mTOR: mammalian target of rapamycin, NF-κB: nuclear factor kappa-light-chain-enhancer of activated B cells, NLRP3: NLR family pyrin domain containing 3, NO: nitric oxide, ROCK2: Rho Associated Coiled-Coil Containing Protein Kinase 2, ROS: reactive oxygen species, SOD: superoxide dismutase, TGF-β: transforming growth factor-β, TNF-α: tumor necrosis factor-alpha, 6-OHDA: 6-hydroxydopamine.
